# Pain after vaginal prolapse repair surgery with mesh is a post‐surgical neuropathy which needs to be treated – and can possibly be prevented in some cases

**DOI:** 10.1111/ajo.12804

**Published:** 2018-03-25

**Authors:** Thierry Vancaillie, Yasmin Tan, Jason Chow, Lauren Kite, Liz Howard

**Affiliations:** ^1^ Department of Women's and Children University of New South Wales Sydney New South Wales Australia; ^2^ Royal Hospital for Women Sydney New South Wales Australia; ^3^ Women's Health & Research Institute of Australia Sydney New South Wales Australia; ^4^ Vulval Health Clinic Royal Hospital for Women Sydney New South Wales Australia; ^5^ Chronic pelvic pain clinic Royal Hospital for Women Sydney New South Wales Australia; ^6^ Osteopath, Pain Management, Education and Support Women's Health & Research Institute of Australia Sydney New South Wales Australia

**Keywords:** chronic pain, mesh, pelvic organ prolapse, post‐surgical neuropathy, treatment algorithm

## Abstract

Post‐surgical neuropathy leading to chronic pain is a recognised complication. It also can occur after surgery for pelvic organ prolapse repair involving mesh. Post‐surgical neuropathy needs to be identified and properly treated to minimise the occurrence of chronic pain. A treatment algorithm is put forward for discussion .

2017 was the Global Year against pain after surgery as per the International Association for the Study of Pain. Chronic post‐surgical pain is to be included in the next International Classification for Diseases (ICD‐11). Any surgery can lead to persistent post‐operative pain, which we now define as ‘post‐surgical neuropathy’. It is clinically expressed by immediate, intense, neuralgic‐quality pain, or a persistence of pain beyond the normal tissue healing time. Lavand'homme^1^ estimates that post‐surgical neuropathy occurs in up to 35% of cases.

Certain procedures have well‐documented incidences of post‐operative neuropathies, such as for instance open heart surgery^2^ or breast surgery. In our specialty, the issue of persistent post‐operative pain also exists. Brandsborg conducted a retrospective study of 1135 women with an incidence of 32% reporting persisting pain four months after hysterectomy (abdominal and vaginal). The same author also conducted a prospective study in 90 patients, showing with objective testing, that chronic pain is present in 17% of patients at four months.^3^ The incidence of post‐operative neuropathy after caesarean section is estimated to be approximately 10%. Nikolajsen first reported that 27 out of 220 (12.3%) women complained of abdominal pain three months after caesarean section, with 13 of them (5.9%) being significantly affected on a daily basis.^4^ The natural history of post‐surgical neuropathy is variable and poorly documented in the literature. Some patients improve over the course of a couple of years, with others continuing to struggle.^5^

The main risk factors for developing post‐surgical chronic pain are: (i) pre‐existing pain and (ii) inadequate post‐operative pain control.^2^, ^3^, ^4^ Both these factors can and should be addressed when treating patients with pelvic organ prolapse.

Surgeons are ill‐equipped to deal with chronic pain, mainly because they will rarely see patients beyond a couple of weeks after the intervention. If they do, they will often not know what approach to take. It is essential for the surgeon to recognise the early signs and symptoms of nerve trauma and to address the issues, in order to avoid chronic pain for these women.

Post‐surgical pain is not necessarily due to a botched operation and surgeons should not feel defensive when a patient returns with complaints of pain. Moreover, in cases of immediate and severe pain, the most appropriate treatment may be surgical revision, to allow for the best chance of pain resolution.^6^

Complications after prolapse repair surgery in women involving mesh have been extensively reported recently in the mainstream media as well as various social media outlets. Due to the severity of the complications and the suffering the women have endured, it is appropriate to briefly review the pathophysiology of this particular condition.

Surgery is in most cases a trauma involving predominantly only the soft tissues. There is vascular injury, transection of muscle and collagen fibres as well as terminal nerve fibres. During the healing phase the body will initiate neo‐vascularisation, will remodel the collagen and the muscle fibres – nerve fibres will sprout and reconnect with their targets.^7^ This is adaptive healing, quickly achieved in the young and a bit slower as we age. The presence of a foreign object in a wound is common, not the least through the use of suture material. Foreign bodies are isolated by the arrival of giant cells and subject to an inflammatory reaction resulting in either removal or incorporation of the object. Giant cells will remain in place as long as the foreign body is present. If the patient has a systemic infection at the time of implant or soon after, such as tooth decay, the foreign body can act as a focus for a secondary infectious site, which may remain sub‐clinical for many years, leaving patients feeling unwell, or may lead to formation of a fistulous tract. The size of the mesh pores and the vaginal flora have been discussed as potential contributing factors to the relatively high number of infections associated with insertion of trans‐vaginal mesh.^8^

Healing can become maladaptive if function of the pelvic organs, pelvic girdle and soft tissues is not restored soon after the surgical intervention. Chronic pain is a frequent complaint resulting from maladaptive healing. The aim of this publication is to focus on prevention and treatment of persistent post‐surgical pain in women undergoing mesh implants for the treatment of pelvic organ prolapse.

Prevention of chronic pain post‐surgery involves a two‐prong approach, namely identifying and possibly treating pre‐existing pain issues and risk factors for developing chronic pain, and secondly, appropriately address pain at the time of surgery and the immediate aftermath. This goes beyond leaving a script for opioids or other medications. Promoting early ambulation among other non‐pharmaceutical means for instance, is essential.

## Before surgery

Pelvic organ prolapse is not a trigger for pain. Induced pain at the time of physical examination of a patient with pelvic organ prolapse should not be attributed to the prolapse itself except in rare cases. Women may complain of discomfort, mainly low back pain, after a long day on their feet – that's not the same as complaining of perineal pain. Differentiating pelvic discomfort from true perineal pain is an essential clinical element in the proper selection of patients. A patient complaining of perineal pain should be offered treatment for the pain, prior to contemplating any type of surgical intervention for a prolapse which may co‐exist. This underwrites the importance of a properly conducted clinical interview and examination.

The presence of pre‐existing pain is listed as a major predisposing factor in the pathophysiology of chronic pain by most authors.^9^ It is therefore crucial that the surgeon be cognisant and knowledgeable of perineal pain conditions.

## At the time of surgery

An important aspect in the prevention of chronic pain is adequate pain management at the time of surgery and during the days and weeks thereafter.^2^ The key to pain control in the immediate hours post‐intervention involves liberal use of local anaesthesia intra‐operatively, and some form of patient‐controlled postoperative analgesia. In most hospitals, the anaesthesiologist is responsible for pain management during the first 24 h after the procedure. Patients typically stay a day or two in hospital and are then discharged with a script for oxycodone or similar narcotic. Post‐surgical appointments are scheduled for several weeks later. This creates a ‘no‐man's land’ of pain management for the patient. Who should they turn to when the oxycodone isn't holding the pain down? Adequate instructions, whichever form that takes, are required. Ideally, the patient's general practitioner takes the lead in this endeavour. As stated before, the most vulnerable patients are those who were suffering from perineal or pelvic pain prior to the prolapse repair surgery. Even if these patients have an adequate pain management plan in place, there will be a need for close monitoring and – in most cases – adaptation of the plan.

## Follow‐up appointment(s)

**Figure 1:**
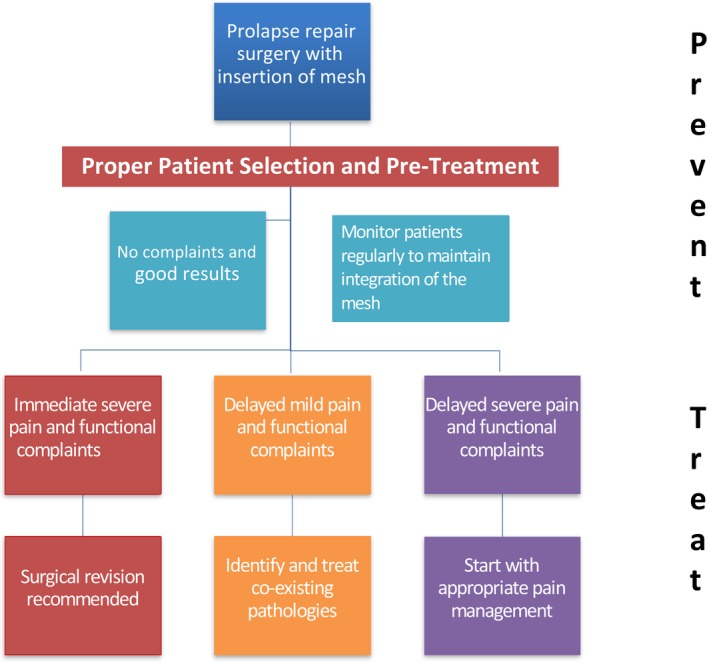
Treatment algorithm for patients with post‐surgical neuropathy post mesh surgery.

Many patients complain that there was no review of pain management during the follow‐up consultation with their specialist. The review is often narrowly focused on the anatomic result of the surgical correction. And complaints of pain are commonly disregarded or discounted with a pat on the back and words to the tune of ‘give it some time’. The issue with post‐surgical neuropathy is that valuable time has already passed and action is required now. A treatment algorithm for pain management is summarised in Figure 1.

## Regular monitoring

Prevention of chronic pain syndromes after vaginal mesh surgery requires regular monitoring. In a recent publication, Milani *et al*. note that the mesh erosion rate increases over time, from 17% at one year to 42% at seven years. The study also highlights the fact that nearly half the women with mesh erosion remained asymptomatic. This underwrites the complex relationship between the presence of a trigger for pain and the clinical expression of pain in the individual patient.^10^ The perineum and vagina are unique in that the tissues have a specific ageing path secondary to their dependence on oestrogen. Having dealt with post‐surgical neuropathy in a large number of patients after vaginal mesh surgery, it has become apparent that some will present with a pain syndrome many years after their sentinel surgical procedure, commonly during peri‐menopause or shortly after menopause. This illustrates the important interplay between hormonal environment, neural and possibly immune systems. In our experience – and curiously the topic of menopause remains silent in most publications on complications of mesh surgery – simple re‐institution of hormone replacement is often not sufficient to stop and reverse the pain. A holistic, interdisciplinary approach is required, incorporating all aspects of the woman's health. We need to identify and treat pain as early as possible in the process. This involves active questioning of the patient about any discomfort with intercourse for instance, as well as a regular physical examination to investigate whether gentle probing of the vaginal wall covering the mesh causes tenderness. Treatment of incipient vaginal wall pain might be achieved using topical amitriptyline with or without topical oestriol to maintain maturation. We use a proprietary compounded preparation (Stenlake, Sydney, New South Wales, Australia) containing low‐dose amitriptyline and oestriol, which appears to be well tolerated on the vulval as well as vaginal skin. The most commonly prescribed compounded amitriptyline is a ‘2% in VersaBase (PCCA Compounding, Houston, TX, USA) – 100 mg’ preparation. The patient applies a small amount to the painful area once or twice a day.

## Immediate, severe post‐surgical pain

Severe post‐surgical pain which starts in the recovery room, needs to be urgently addressed. Severe pain implies that the surgery has resulted in more than average soft tissue trauma and involves the peripheral nervous system.^7^ Neuropathic pain should be identified early as the therapeutic approach varies from that of simple soft tissue trauma. The use of validated tools such as the Douleur Neuropathique 4 (DN4) can guide questioning and examination to assess the likelihood of neuropathic pain. Severe acute pain related to nerve trauma in surgery needs to be swiftly addressed, as there is a real risk for persistent pain due to neural changes locally at the dorsal root ganglion, as well as centrally. If the patient complains of pain despite infiltration of local anaesthetic at the time of surgery, there should be suspicion of a major issue, which needs urgent attention. In the absence of complications such as a haematoma, one suspects that the surgical trauma has affected a larger branch of one of the nerves of the pelvis or perineum. Complete transection of a neural branch would result in numbness rather than pain, so transection is likely not the reason for the severe pain in the recovery room. Rather, the nerve is stimulated by a process of compression or elongation. Effects of compression and elongation are secondary to the use of sutures and insertion of foreign objects such as mesh. It is therefore logical to consider removing the mesh(es) in its entirety as well as the sutures keeping it in place.^6^ The window of opportunity to remove the mesh before damage is irreversible is unknown – but it is reasonable to set a 24 h limit for the decision‐making process, which leaves time to complete emergency imaging tests if required and involve colleagues for a second opinion.

## Pain management in the early weeks post‐surgery

Pain management in the early days and weeks after surgery is aimed at treating the soft tissue trauma and based predominantly on the use of anti‐inflammatory as well as narcotic medications (Table 1. However, physical elements such as ice or heat packs and low‐impact exercises are also important. The most effective allied health adjunctive treatment in the immediate aftermath of surgery is acupuncture, thought to be a form of neuro‐modulation .^11^

**Table 1:** Pain management in the early days and weeks post‐surgery

1	Anti‐inflammatory medications (consider rectal suppositories)
2	Injection of local anaesthetic (addition of cortisone is of uncertain value)
3	Topical oestrogen is resumed as soon as possible
4	Pain medication (tramadol, tapentadol, buprenorphen) – avoid codeine for its significant constipating effect, which will negatively impact pain management
5	Anti‐neuropathic medications (tricyclic antidepressants, gabapentinoids, selective noradrenaline reuptake inhibitors)
6	Peripheral and regional blocks (caudal, pudendal)
7	Allied health in a supportive role

As time goes on and pain persists, it is advisable to turn attention to the use of medications immediately impacting the transmission of the neural stimulus. The most effective medications are the so‐called membrane stabilisers, such as local anaesthetics. It is not common practice or tradition to proceed with regional blocks, most likely because those who would recommend and perform the blocks are no longer involved in the management of the patient. However, the use of pudendal nerve and caudal blocks make a lot of sense, certainly for those patients who are not making any progress using a more established medical approach. It should be said at this stage that the traditional strong narcotic medications such as oxycodone have less and less a place in pain management as time goes by. When does one switch to one of the ‘weak’ products (tapentadol, buprenorphen, tramadol)? The clinician treating the patient is best placed to make the call. Experience will bring suspicion that a patient will need longer than expected pain management. Rather than renewing the oxycodone script, it might be an opportunity to switch.

## Pain management in the months post‐surgery

When the three‐month threshold is reached, the use of anti‐neuropathic medications, if tolerated, becomes the mainstay of medical management (Table 2). Topical amitriptyline is no doubt a valuable element in the medium‐ to long‐term management of post‐surgical neuropathy in the perineal region. Combination of topical and parenteral pharmaceuticals needs to be titrated to their optimal effectiveness with the lowest possible side‐effect profile.

**Table 2:** Pain management 12 + weeks post‐surgery

1	Anti‐neuropathic medication (tricyclic antidepressants, gabapentinoids, selective noradrenaline reuptake inhibitors)
2	Pain medication (tramadol, tapentadol, buprenorphen)
3	Hormone replacement therapy to be continued
4	Local and regional anaesthetic blocks
5	Psychotherapy: mindfulness – meditation – self‐hypnosis
6	Physiotherapy: transcutaneous electrical nerve stimulation – botulinum toxin infiltrations
7	Osteopathy: manipulation to address associated musculoskeletal dysfunction

Allied health support, such as psychotherapy, physiotherapy and osteopathy become more interventional as opposed to supportive. Treatment of post‐surgical neuropathy is tedious and takes several months. Frustration is common. Patients need to be encouraged to persevere. Treatment is recommended to continue a few weeks beyond resolution of symptoms and if symptoms recur, treatment is resumed as soon as feasible.

A few patients will need more aggressive pain management, even after complete removal of the mesh and suture material. The modalities used, collectively termed neuro‐modulation, are beyond the scope of this dissertation. However, it might be said that referral to a pain medicine specialist for more comprehensive management should not be delayed when patients are not making real progress in returning to normal daily functioning.
